# MicroRNA-645, up-regulated in human adencarcinoma of gastric esophageal junction, inhibits apoptosis by targeting tumor suppressor IFIT2

**DOI:** 10.1186/1471-2407-14-633

**Published:** 2014-08-29

**Authors:** Xiaoshan Feng, Ying Wang, Zhikun Ma, Ruina Yang, Shuo Liang, Mengxi Zhang, Shiyuan Song, Shuoguo Li, Gang Liu, Daiming Fan, Shegan Gao

**Affiliations:** Oncology Department of the First Affiliated Hospital of Henan, University of Science and Technology, No. 24 Jinghua Road, Luoyang, Henan China; State Key Laboratory of Cancer Biology and Xijing Hospital of Digestive Diseases, Fourth Military Medical University, Xi’an, Shaanxi China

**Keywords:** Adencarcinoma of gastric esophageal junction, microRNA-645, IFIT2, Apoptosis

## Abstract

**Background:**

An increasing body of evidence indicates that miRNAs have a critical role in carcinogenesis and cancer progression; however, the role of miRNAs in the tumorigenesis of adencarcinoma of gastric esophageal junction (AGEJ) remains largely unclear.

**Methods:**

The SGC7901 and BGC-823 gastric cancer cell lines were used. The expressions of miR-645 and IFIT2 (Interferon-induced protein with tetratricopeptide repeats 2) were examined by qRT-PCR, The expressions of IFIT2 was examined by western blotting and immunohistochemistry assay. The cell apoptosis was determined by FACS. MiR-645 inhibitor, mimics and plasmid-IFIT2 transfections were performed to study the loss- and gain-function. Caspase-3/7 activity was examined by caspase-3/7 assay.

**Results:**

In the present study, we have reported an increased expression of miR-645 in AGEJ clinical specimens compared with paired non-cancerous tissues. We also observed a significant miR-645 up-regulation in two gastric cancer (GC) cell lines, SGC7901 and BGC-823, which were used as cell models because there was no available AGEJ cell lines established to date. We found that inhibition of miR-645 could sensitize dramatically SGC7901 and BGC-823 cells to both serum starvation– and chemotherapeutic drug–induced apoptosis by up-regulating IFIT2, a mediator of apoptosis via a mitochondrial pathway, with a potential binding site for miR-645 in its mRNA’s 3′UTR. Further investigation exhibited that IFIT2 expression decreases in SGC7901 and BGC-823 cells and AGEJ tissues. IFIT*2* ectopic expression leads to promotion of cell apoptosis, indicating that IFIT2 may function as a suppressor in the development of AGEJ. Furthermore, inhibition of miR-645 induces up-regulation of IFIT2 and increased caspase-3/7 activity compared with control groups.

**Conclusions:**

Our data suggest that miR-645 functions as an oncogene in human AGEJ by, at least partially through, targeting IFIT*2*.

**Electronic supplementary material:**

The online version of this article (doi:10.1186/1471-2407-14-633) contains supplementary material, which is available to authorized users.

## Background

Recent studies have suggested that adencarcinoma of gastric esophageal junction (AGEJ) is distinct from that of distal stomach, with different risk factors, tumor characteristics, and biological behavior [1–4]. Moreover, the incidence of AGEJ has been increasing over the past 30 years, especially in United States and north China [5–9].

microRNAs (miRNAs) are a group of endogenously expressed, non-coding small RNAs, 20–25 nucleotides in length, which are known to negatively regulate gene expression through suppressing translation or decreasing the stability of mRNAs by directly binding to the 3′-untranslated region (3′-UTR) of target mRNAs [10, 11]. Accumulating evidence indicates that miRNAs have important roles in regulating physiological and pathological processes, including development [12], metabolism [13], cell proliferation [14], differentiation [15] and apoptosis [16]. In addition, aberrant post-transcriptional regulation of mRNAs by miRNAs is related with tumorigenesis [17, 18]. The abnormal expression profiles of miRNAs have been reported to be detected in various types of human tumors including lung [19], breast [20], prostate [21], liver [18], colon [22] and gastric cancer [23]. Moreover, some miRNAs can act as oncogenes [24–26] or tumor supressors [27, 28] by regulating the expression of their target genes which have important roles in some key pathways involved in cell cycle progression, apoptosis or proliferation. miRNAs down-regulated in tumour specimens such as miR-22 [29, 30], miR-101 [31, 32], and miR-7 [33, 34] usually function as suppressive miRNAs, while miRNAs upregulated in tumour specimens such as miR-17 [35, 36], and miR-21 [37, 38] usually exert oncogenic roles. These studies suggest that dysregulation of miRNAs is frequently involved in carcinogenesis and cancer progression.

A recent study has indicated that miR-645 may exert the tumor suppressor role in advanced serous ovarian cancer for miR-645 is negatively associated with overall survival of it [39]. In the present study, we found that miR-645 expression was significantly increased in AGEJ clinical specimens compared with paired non-cancerous tissues using microRNA chips. However, the role of miR-645 in the tumorigenesis of AGEJ has not been studied yet. Further study showed that miR-645 was also significantly up-regulated in two gastric cancer (GC) cell lines, SGC7901 and BGC-823, which were used as alternative cell models in the present study. Inhibition of miR-645 in SGC7901 and BGC-823 cells significantly suppressed the apoptosis of SGC7901 and BGC-823 cells in the condition of serum starvation or chemotherapeutic drug by up-regulating IFIT2, a mediator of apoptosis, with a potential binding site for miR-645 in its mRNA’s 3′UTR. The expression pattern of miR-645 and IFIT2 in AGEJ clinical samples were negatively correlated, further suggesting that *IFIT2* is a target gene of miR-645. Moreover, inhibition of miR-645 results in increased caspase-3/7 activity, which is activated by IFIT2. In this study, we investigated whether miR-645 is up-regulated in human adencarcinoma of gastric esophageal junction and inhibits apoptosis by targeting tumor suppressor IFIT2.

## Methods

### Ethics statement

For tissue samples, written informed consent was obtained from patients. The procedures used in this study were approved by the Institutional Review Board of the Henan University of Science and Technology and was conformed to the Helsinki Declaration, and to local legislation.

### Cell lines and culture conditions

Gastric cancer cell lines SGC-7901, BGC-823 and immortalized normal gastric epithelial cell line, GES-1 were kindly bestowed by Prof. Daiming Fan. All the cell lines were maintained in our institute according to recommended protocols. Cells were cultured in RPMI-1640 medium (Invitrogen, Carlsbad, CA, USA) supplemented with 10% fetal bovine serum (FBS) (Invitrogen, Carlsbad, CA, USA) at 37°C in a 5% CO2 incubator.

### Human specimens

All experimental procedures were approved by the Institutional Review Board of the Henan University of Science and Technology. Written informed consent was obtained for all patient samples. Human AGEJ specimens (n = 43) and patient paired non-cancerous specimens were obtained from patients at the first affiliated hospital, Henan University of Science and Technology, with informed consent from each patient.

### RNA purification, cDNA synthesis, and quantitative real-time PCR (qRT-PCR)

Total RNA of cultured cells was extracted with TRIzol reagent (Invitrogen, Carlsbad, CA, USA) according to the manufacturer’s protocol and RNAs were stored at −80°C before qRT-PCR analysis. Mature miR-645 expression was detected using a mirVana TM qRT-PCR miRNA Detection Kit (Ambion Inc. Austin, Texas), with U6 as an internal control. IFIT2 expression was detected with primers F: 5′AGCGAAGGTGTGCTTTGAGA 3′, R: 5′GAGGGTCAATGGCGTTCTGA3′ (product length: 125 bp; Tm: 60°C; GC%: F-50%, R-55%; start-end: 643-748 bp) and GAPDH was used as an internal control. PCR products were separated on an ethidium bromide-stained 1.5% agarose gel and visualized with UV.

### Cell transfection

The human miR-645 duplex agomir (400 nM), antagomir (400 nM) and negative control were designed and provided by Ribobio (Guangzhou, Guangdong, China). Plasmid-IFIT2 and the negative control plamid were purchased from Ribobio Inc (Guangzhou, Guangdong, China).

### miRNA target prediction

To find potential miRNA target genes, TargetScanHuman website (http://www.targetscan.org/) was used, the binding free energy was calculated and biding sites were analyzed using http://bibiserv.techfak.uni-bielefeld.de/rnahybrid website.

### Vector constructs and luciferase reporter assay

To construct IFIT2-3′UTR plasmid, a wild-type 3′-UTR fragment of human IFIT2 mRNA (1226–1233 nt, Genbank accession no. NM_001547.4) containing the putative miR-645 binding sequence was amplified by RT-PCR and cloned into the site between Xho I and Not I downstream of the luciferase reporter gene of the psiCHECK™ vector (Promega, USA). A mutant of the single miR-645 binding site (5′- AGCCTAG −3′ to 5′- TCGGATC −3′) in the 3′-UTR of IFIT2 was included by Site-Directed Mutagenesis Kit (SBS Genetech, Beijing, China). Wild and mutant types of pmirGLO-IFIT2-UTR vectors were validated by DNA sequencing.

The nucleotide sequences of primers for IFIT2-3′UTR (WT) clone:

IFIT2XhoIF2: 5′CCGCTCGAG AGAATAGAGATGTGGTGCCCACTAGGCTACTGCTG 3′.

IFIT2NotIR2: 5′ATAAGAATGCGGCCGC TTAAAATGGAATCAGTGACTTTTATTTCTCATAACAGAG 3′.

The nucleotide sequences of primers for IFIT2-3′UTR (MT) clone:

mutIFIT2F2: 5′TTCTAGGTAGATGCTGAATTCGGATCACATCAAAGTTGGTGTGAAC 3′.

mutIFIT2R2: 5′GTTCACACCAACTTTGATGTGATCCGAATTCAAGEJTCTACCTAGAA 3′.

Cells were transfected with the miR-645 mimics, NC and pmirGLO plasmid in 24-well plates using lipofectamine™ 2000 (Invitrogen) according to the instructions. 48 h later, cells were harvested and analyzed for luciferase activity using the Dual-Luciferase Reporter Assay System (Promega, USA) and detected by the GloMaxTM 20/20 detection system (E5331, Promega).

### caspase-3/7 assay

The activity of caspase-3 and caspase-7 was detected in 96-well format (2 × 10^3^ cells/well) using the Caspase-Glo 3/7 Assay (Promega) according to the instructions. 100 μL Caspase-Glo 3/7 reagent were supplemented into each well and then incubated at room temperature for 1 h follwong the luminescence was detected using the M200 microplate fluorescence reader (Tecan). The background luminescence associated with cell culture and assay reagent (blank reaction) was subtracted from experimental value.

### MTT assay

Cells were transfected with 100 nM miR-645 inhibitor (Genepharma, Shanghai, China), mimics (Ribobio Inc., Guangzhou, Guangdong, China) or 100 nM plamid-IFIT2 (Ribobio Inc., China). Twenty-four later, cells were seeded in 96-well plates (2 × 10^3^/well). The viability of cells was examined by MTT (3–2, 5-diphenyl tetrazolium bromide) assay (Sigma, USA) according to instructions at designated time.

### Western blotting

Total protein from cultured cells were lysed by Lysis Buffer containing PMSF on ice. Then protein were electrophoresed through 12% SDS polyacrylamide gels and were then transferred to a PVDF membrane (Millipore). Membranes were blocked with 5% non-fat milk powder at room temperature for 1 h and incubated overnight with primary antibodies. Membranes were incubated with secondary antibodies labeled with HRP for 1 h at room temperature after three 10 min washes in TBS-T (triethanolaminebuffered saline solution with Tween). Finally, the signals were detected using ECL kit (Pierce Biotech., Rockford, IL, USA) and the membranes were scanned and analyzed using a Bio-Rad ChemiDoc XRS + imaging system with imaging software (version quantity 1). The protein expression was normalized to an endogenous reference (Tubulin) and relative to the control. The Spectra multicolor broad-range protein ladder (Fermentas) was used as molecular marker. All the antibodies used in western blot assay are listed in Additional file 1: Table S1.

### Immunohistochemistry and immunohistochemical scoring

Paraffin sections, 4-μm in thickness, were baked for 2 h at 65°C and deparaffinized. Antigen retrieval was performed using citrate sodium buffer (PH 7.2) at 95°C for 15 minutes and then slides were cooled at room temperature for 30 minutes. After being treated with 3% hydrogen peroxide for 15 minutes to block the endogenous peroxidase, the sections were treated with normal goat serum confining liquid for 30 minutes to reduce non-specific binding and then rabbit polyclonal anti-IFIT2 (1:500, HPA003408, Sigma-Aldrich. Shanghai, China) was incubated the sections for 12 h at 4°C. After rewarming for 1 h and washing for 5 times, sections were incubated with secondary antibody for 30 minutes at room temperature. Diaminobenzidine (DAB) was used for color reactions. Subsequent immunohistochemical staining was scored as previously described [40].

### Statistical analysis

Data were expressed as Mean ± SD of three independent experiments. For statistical tests, SPSS statistical software package, version17.0 (SPSS, Chicago, IL, USA) was used. The student’s *t*-test, the one-way ANOVA and two-way ANOVA test were performed for relative band density of western blotting and MTT OD values. The correlation between miR-645 and IFIT2 was analyzed with Spearman rank correlation. P values <0.05 were considered statistically significant.

## Results

### Expressions of miR-645 are up-regulated in AGEJ clinical samples

**Figure 1:**
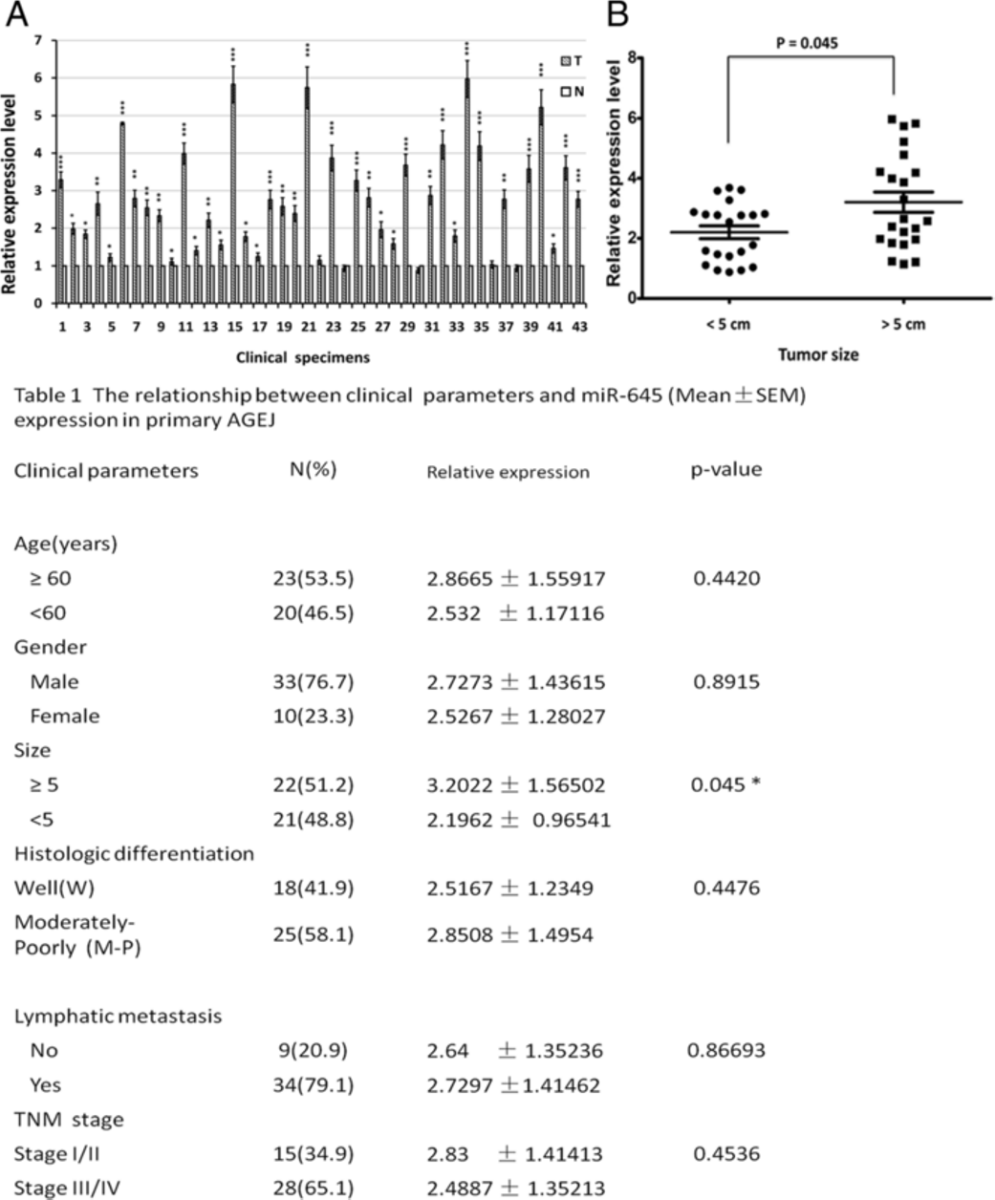
**Expressions of miR-645 are up-regulated in AGEJ clinical samples. A**. expression of miR-645 in 43 human AGEJ clinical samples relative to the adjacent paired normal human gastric cardiac non-cancerous tissues, was measured by quantitative RT-PCR (The values indicate the mean ± SEM, n = 3, *t*-test,* p < 0.05, ** p < 0.01, *** p < 0.001). **B**. comparison of relative expression of miR-645 in human AGEJ clinical samples of different tumor size. (Two-tailed *t*-test,* p < 0.05).

To assess the role of miR-645 in the tumorigenesis of AGEJ, we first used qRT - PCR method to measure miR-645 expression of 43 human AGEJ clinical tissues, and found that miR-645 was significantly up-regulated in AGEJ clinical tissues compared with patient paired gastric cardiac non-cancerous tissues (Figure 1A). To gain further insights into the observation mentioned above, we examined the relationship between miR-645 expression and patients clinical parameters. Analysis showed that miR-645 expression was irrelevant with age, sex, tumor differentiation, lymphnode metastasis and TNM stage (Table 1: The relationship between clinical parameters and miR-645 expression in primary gastric cardia adenocarcinoma), but was in a positive correlation with the tumor size, namely, tumor size greater than or equal to 5 cm group showed significant increased miR-645 expression compared with tumor size less than 5 cm group (Figure 1B & Table 1, *t*-test, *P = 0.045).

**Table 1:** **The relationship between clinical parameters and miR-645 expression in primary gastric cardia adenocarcinoma**

Clinical parameters	N (%)	Relatives expression (Mean ± SEM)	p-value
Age (years)			
≥ 60	15 (50)	2.1286 ± 0.59201	0.568
<60	15 (50)	1.9571 ± 0.52402	
Gender			
Male	18 (60)	2.1111 ± 0.42783	0.462
Female	12 (40)	2.3333 ± 0.65328	
Size			
≥5	13 (43.3)	2.7385 ± 100128	0.004*
<5	17 (56.7)	1.8235 ± 1.52214	
Histologic differentiation			
Well (W)	15 (50)	2.138 ± 0.1866	0.9427
Poorly (P)	15 (50)	2.198 ± 0.1903	
Lymphatic metastasis			
Yes	12 (40)	2.4583 ± 0.77864	<0.001**
No	18 (60)	1.4944 ± 1.36273	
TNM stage			
Stage I/II	16 (53.3)	2.9125 ± 1.33660	<0.001**
Stage III/IV	14 (46.7)	1.4857 ± 0.73991	

(*p < 0.05; **p < 0.001).

### Depletion of miR-645 promotes apoptosis of gastric cancer cells

To investigate the role of miR-645 in the phenotypic characteristics of AGEJ progression, we used two gastric cancer (GC) cell lines, SGC7901 and BGC-823 as cell models. qRT-PCR results showed that miR-645 expression was significantly up-regulated compared with immortalized GC cell line, GES-1 (Additional file 2: Figure S1, *P* < 0.001).

**Figure 2:**
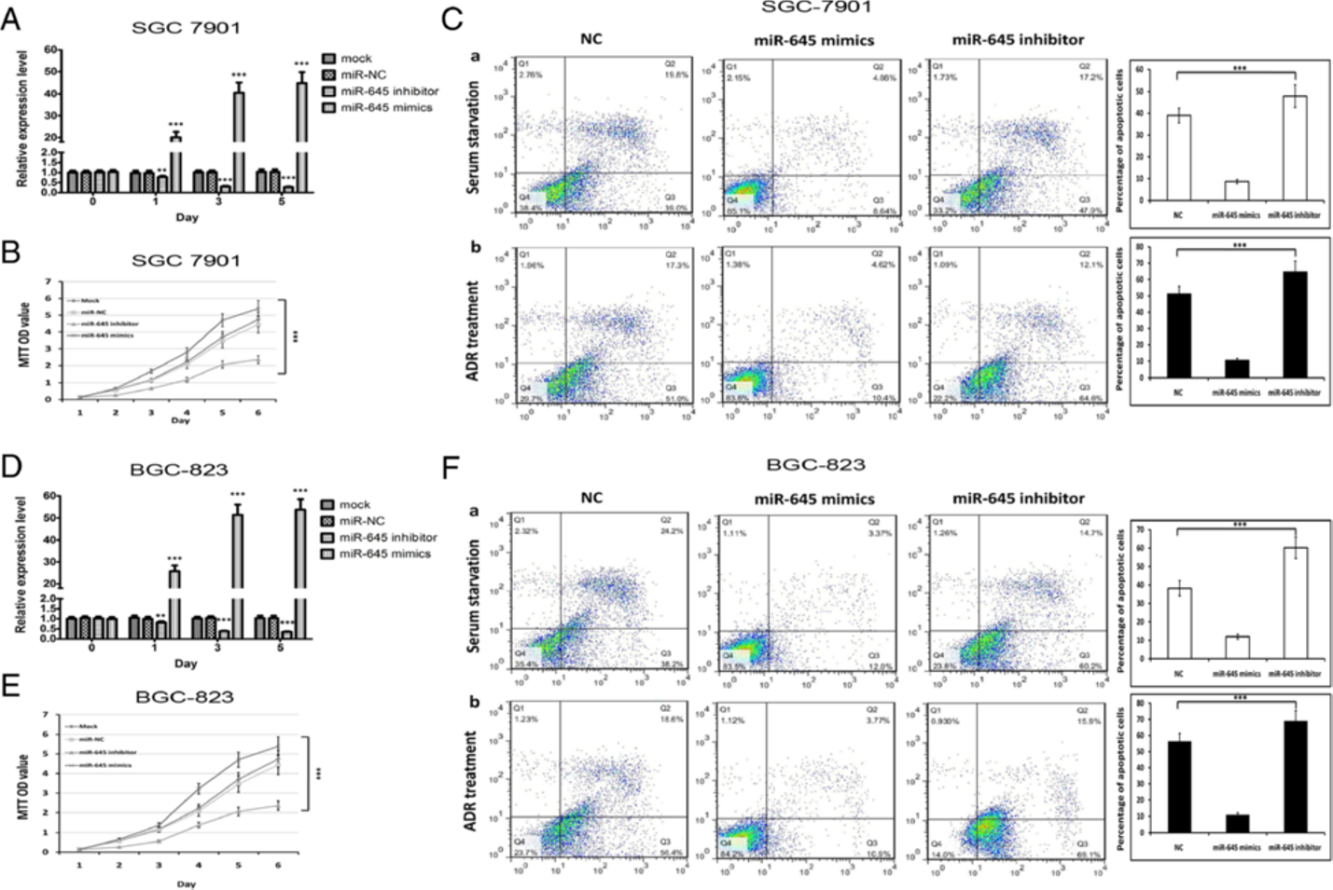
**Depletion of miR-645 promotes apoptosis of gastric cancer (GC) cells. A** &**D**. The level of miR-645 was measured by quantitative PCR at designated time (One-way ANOVA analysis, the values indicate the mean ± SD, Figure 2 **A**, F =426.588, P < 0.001; Figure 2 **D**, F = 685.026, P < 0.001). **B** &**E**. GC cells transfected with miR-645 mimics and inhibitor subjected to MTT assay daily for 6 days (Two-way ANOVA analysis, Figure 2 **B**, F = 52.602, p < 0.001; Figure 2 **E**, F = 42.847, p < 0.001). **C** &**F**. GC cells cells transfected with miR-645 mimics and inhibitor were collected for FACS analysis after 72 h (The values indicate the mean ± SD, n = 3, One-way ANOVA analysis, Figure 2 **C**-a, F = 121.600, p <0.001; Figure 2 **C**-b, F = 250.400, p <0.001; Figure **F**-a, F = 194.815, p <0.001; Figure 2 **F**-b, F =412.741, p <0.001).

SGC7901 and BGC-823 cells were transiently transfected with mature miR-645 mimics, inhibitor, mock transfected, or miR-NC. As shown in Figure 2A and D, quantitative RT-PCR results show that expression of miR- 645 mimics or inhibitors significantly up-regulate or down-regulate the expression level of miR-645, respectively, in SGC7901 and BGC-823 cells from the first to fifth day post transfection (Figure 2A, *P* < 0.001; Figure 2D, *P* < 0.001) compared to NC and mock controls.

SGC7901 and BGC-823 cells transfected with miR-645 inhibitors and mimics showed significantly lower and higher levels of cell proliferation, respectively, comparision with the NC or mock groups in the presence of ADR (0.2 μg/mL) as determined by MTT assay (Figure 2B, *P* < 0.001; Figure 2E, *P* < 0.001).

Anniex Vapoptosis assay exhibited significant increased and decreased apoptosis rates of miR-645 depletion and ectopic expression groups compared with NC groups in the serum-free condition or in the presence of anticancer drug, adriamycin (ADR) (Figure 2C a-b, *P* < 0.001; Figure 2 F a-b, *P* < 0.001).

### IFIT2 is a target of miR-645

**Figure 3:**
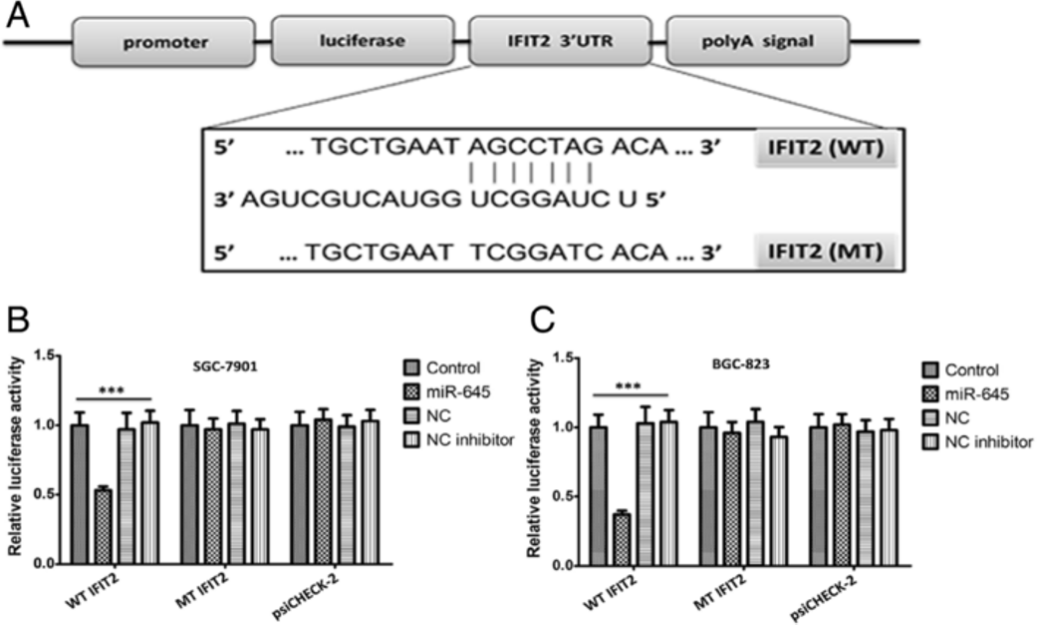
**Validating the predicted binding sites between miR-645 and IFIT2. A**. The schematic diagram shows the construct of Luc-IFIT2 3′UTR and Luc-IFIT2 3′Mut UTR. Both Luc-IFIT2 3′UTR and Luc-IFIT2 3′Mut UTR were cloned into a pmirGLO plasmid downstream of the firefly luciferase coding region between the PmeI and XbaI sites. B&C. SGC7901 cells **(B)** or BGC-823 cells **(C)** were co-transfected with the psiCHECK-2 constructs containing either IFIT2 3′UTR or IFIT2 3′Mut UTR and either the miR-645 inhibitor or the miR-645 mimics for 48 h. Values indicate the relative luciferase activity after normalization to Renilla luciferase activity (The values indicate the mean ± SD, n = 3, One-way ANOVA analysis, Figure 3 **B**, F = 283.244, Figure 3 **C**, F = 143.313. ***p < 0.001).

Previous data suggest that miR-645 might be an oncogene of advanced serous ovarian cancer. Thus we further searched for the potential targets of miR-645 by algorithm of Target Scan Human. Among them, IFIT2, a tumor suppressor, was found to have putative miR-645 binding sites within its 3′UTR (Figure 3A). Then we performed luciferase reporter assay using SGC7901 and BGC-823 cells to verify whether IFIT2 was a direct target of miR-645. Wild-type and mutant IFIT2-3′UTR containing the putative binding site of miR-645 were cloned into psiCHECK-2 vector downstream from luciferase gene (Additional file 3: Figure S2). Introduction of miR-645 reduced the lucirferase activity from the IFIT2 3′UTR reporter vector significantly (Figure 3B, *P* < 0.001; Figure 3C, *P* < 0.001), but did not affect the lucirferase activity from the mutant IFIT2 3′UTR reporter vector, supporting the direct interaction of miR-645 with IFIT2. These results further suggest that miR-645 may suppress the IFIT2 expression by targeting the 3′-UTR of IFIT2 mRNA.

### Expression of miR-645 and IFIT2 are negatively related in AGEJ clinical samples

**Figure 4:**
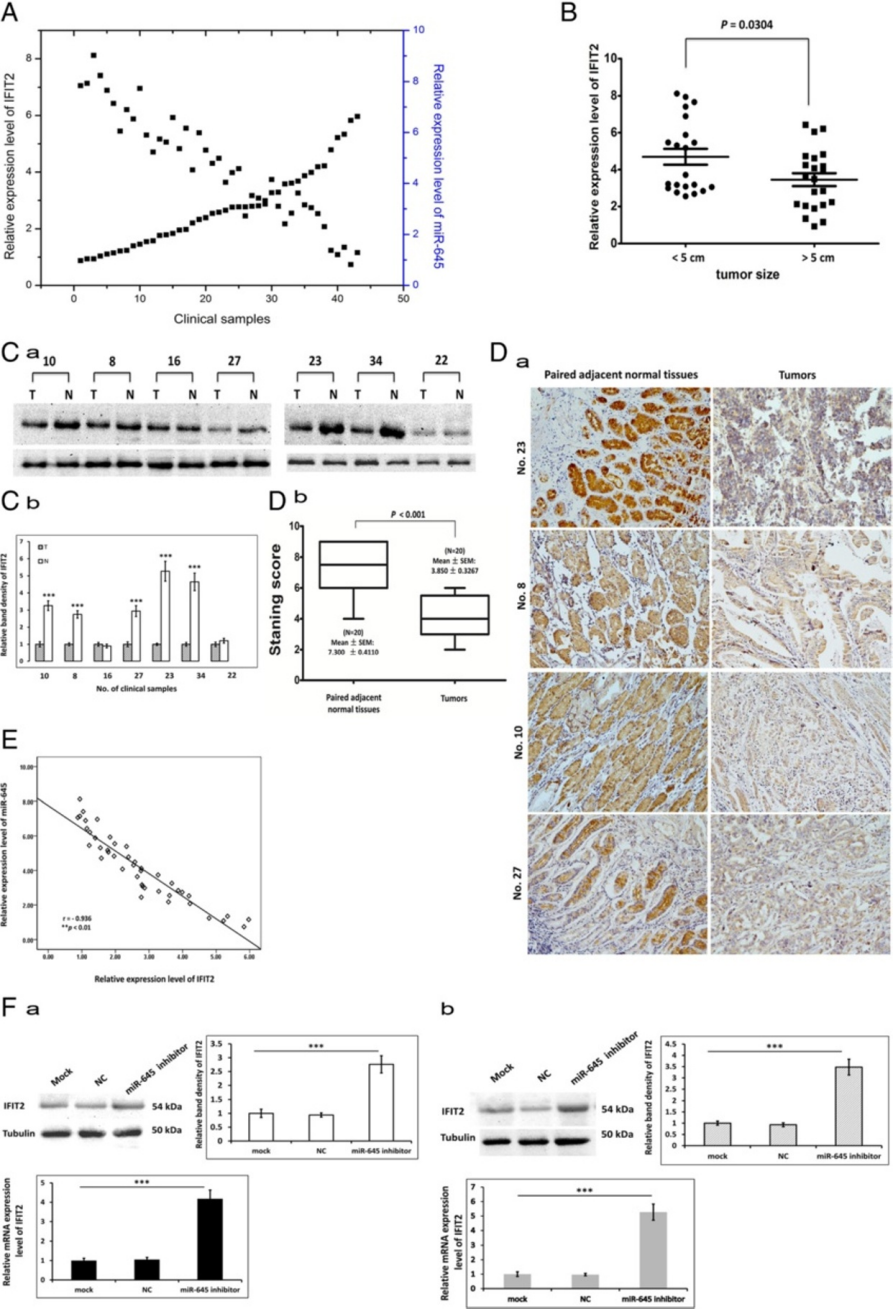
**Expression of miR-645 and IFIT2 negatively correlate in AGEJ clinical samples and IFIT2 was down-regulated in AGEJ tissues compared with paired non-cancerous tissues. A**. Expression of *IFIT2* and miR-645 in AGEJ clinical samples were analyzed by quantitative PCR. **B**. comparison of relative expression of *IFIT2* in human AGEJ clinical samples of different tumor size. (*t*-test, *p < 0.05). **C**. Expression of IFIT2 examined by western blotting (a, b: the values indicate the mean ± SD, normalized to tubulin, n = 3, *t*-test, ***p <0.001) **D**. a. Representative images shown are positive immunohistochemical staining of IFIT2 in human AGEJ specimens and matched adjacent normal tissues (magnification 200×). b. Staining scores of IFIT2 (*t*-test, ***p <0.001). **E**. Scatter plots showing the negative linear correlation between the mRNA expression of *IFIT2* and that of miR-645 in 43 AGEJ clinical samples. **F**. IFIT2 and *IFIT2* expression measured by western blotting in SGC7901(a) and BGC-823 cells (b). (The values indicate the mean ± SD, n = 3, One-way ANOVA analysis, ***p < 0.001).

To further assess the relation between miR-645 and IFIT2, we examined the IFIT2 expression in 43 AGEJ clinical samples using qRT-PCR. It was found AGEJ tissues had a remarkable lower expression level of IFIT2 than the paired non-cancerous tissues (Figure 4A), and IFIT2 expression was inversely correlated with the tumor size (Figure 4B, *P =* 0.0304). Namely, tumor size greater than or equal to 5 cm group showed significant down-regulated IFIT2 expression compared with tumor size less than 5 cm group. To validate the data, we subsequently measured protein expression of IFIT2 using western blotting (Figure 4C a-b), and the results showed a similar pattern to the observations found by qRT-PCR. Immunohistochemistry assay exhibited a significant decreased expression of IFIT2 in AGEJ tissues paired non-cancerous tissues (Figure 4D a-b, *P <* 0.001). Then we analyzed the relationship between miR-645 and IFIT2 expression and found that IFIT2 expression level was negatively correlated with that of miR-645(Figure 4E, *P <* 0.01). Moreover, SGC7901 (Figure 4F a) and BGC-823 (Figure 4F b) cells transfected with miR-645 inhibitor have significantly increased IFIT2 expression at the protein and mRNA levels compared to mock and NC groups (Figure 4F a-b, *P <* 0.01). Our findings indicate that miR-645 may directly regulate the expression of IFIT2.

### IFIT2 mediates the function of miR-645 by promoting SGC7901 and BGC-823 cells apoptosis

**Figure 5:**
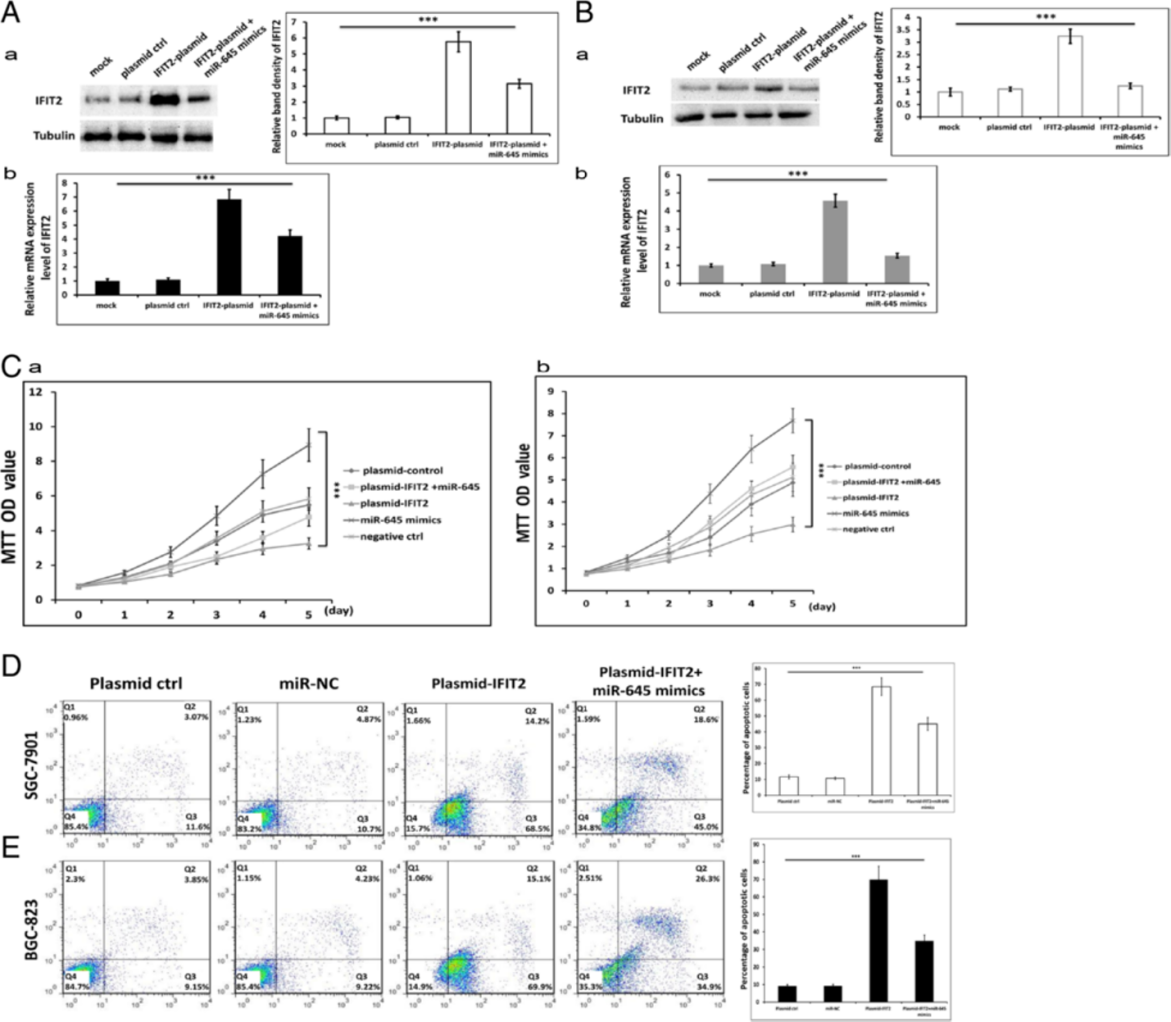
**IFIT2 mediates the function of miR-645 by promoting GC cell apoptosis. A** &**B**. Expression of IFIT2 examined by western blotting (**A**: SGC7901; **B**, BGC-823. a, normalized to tubulin, the values indicate the mean ± SD, One-Way ANOVA analysis, for SGC7901, F = 189.307, for BGC-823, F = 85.374; b, The values indicate the mean ± SD, One-Way ANOVA analysis, for SGC7901, F = 85.374, for BGC-823, F = 219.921; ***p < 0.001). **C**. SGC7901 (a) and BGC-823 (b) cells subjected to MTT assay daily for 6 days (the values indicate the mean ± SD, Two-way ANOVA analysis, for SGC7901, F = 13.768, for BGC-823, F = 16.409, ***p <0.001). **D** &**E**. SGC7901 **(D)** and BGC-823 **(E)** cells were collected for FACS analysis after 72 h in the presence of ADR (0.05 μg/mL) (the values indicate the mean ± SD, n = 3, One-Way ANOVA analysis; for SGC7901, F = 361.749, for BGC-823, F = 229.952; ***p < 0.001).

To confirm that the induction of cell apoptosis in SGC7901 and BGC-823 cells by miR-645 was mediated by targeting *IFIT2*, we performed *IFIT2* plasmid transfection in SGC7901 (Figure 5A) and BGC-823 (Figure 5B) cells to up-regulate the expression of IFIT2. Western blotting and quantitative PCR showed that up-regulation of IFIT2 by *IFIT2* plasmid transfection could be decreased significantly by miR-645 mimics. MTT assay showed that cells transfected with plasmid-IFIT2 proliferation was suppressed, however, cells transfected with miR-645 mimics proliferation increased significantly compared with plasmid-control and plasmid-IFIT2 + miR-645 groups (Figure 5C a-b, *P <* 0.001). Furthermore, introduction of IFIT2 promoted apoptosis of SGC7901 and BGC-823 cells and miR-645 mimics reduced apoptosis of cells induced by IFIT2 up-regulation compared with NC group in the presence of ADR (Figure 5E-F, *P <* 0.001). Our findings suggested that IFIT2 mediated the function of miR-645 in inhibiting SGC7901 and BGC-823 cells proliferation and inducing cell apoptosis.

### Depletion and up-regulation of miR-645 altered the caspase-3/7 activity

**Figure 6:**
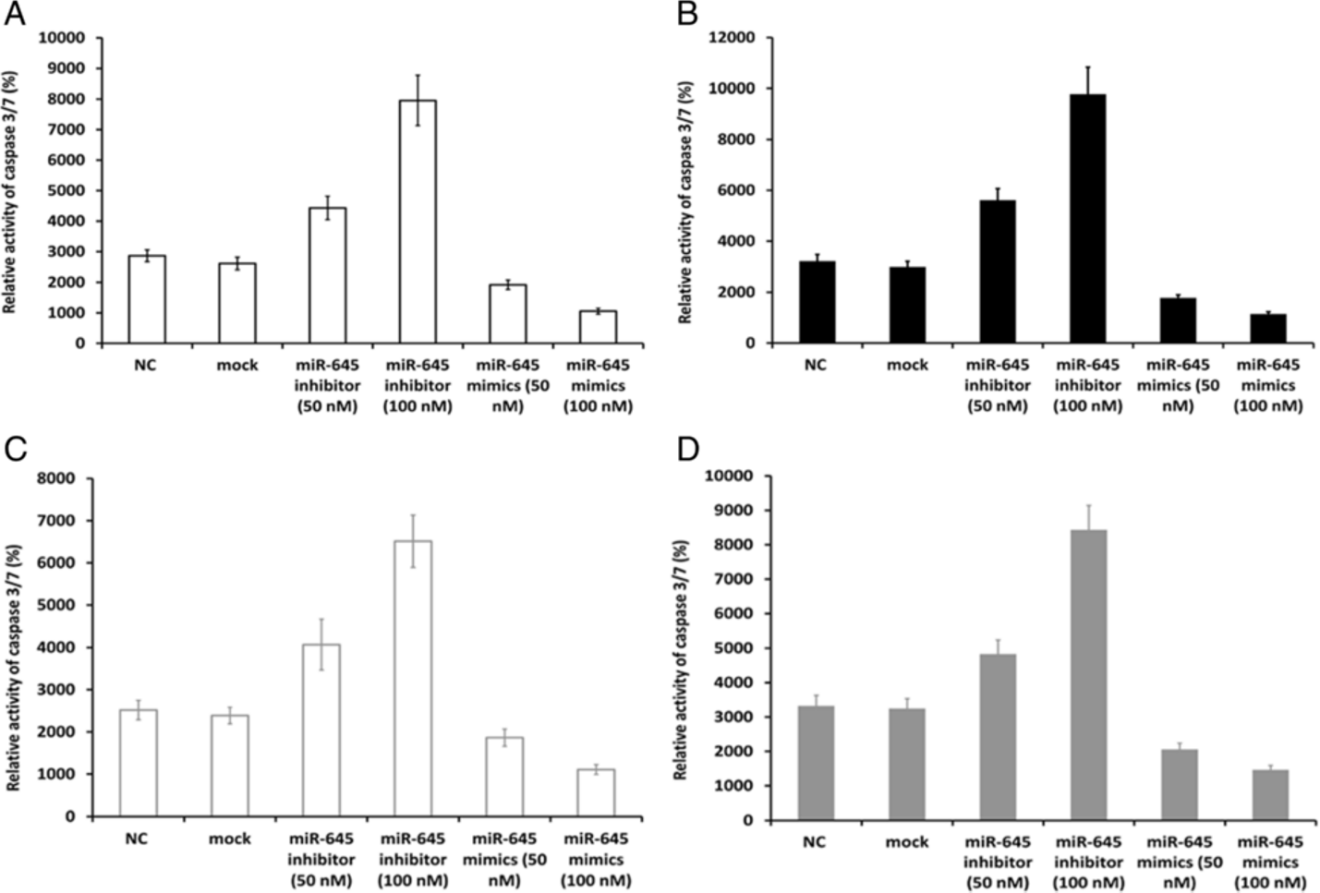
**Caspase-3/7 activity.** Anti-apoptotic ability of SGC7901 cells and BGC-823 cells after exposure to ADR (0.2 μg/mL) or serum starvation was evaluated by caspases-3/7 activity. **A** &**C**, SGC7901 cells; **B** &**D**, BGC-823 cells. (the values indicate the mean ± SD, n = 3, One-Way ANOVA analysis; for **A**, F = 183.930, for **B**, F = 1093.797; for **C**, F = 1861.50, for **D**, F = 1483.604, ***p < 0.001).

IFIT2 has been reported to be a tumor suppressor via mediating cell apoptosis through activating caspase-3/7 activity. Caspase-Glo 3/7 assay showed that miR-645 depletion significantly up-regulated, while miR-645 overexpression down- regulated the caspase-3/7 activity compared with mock and NC groups in the presence of ADR (0.2 μg/mL) (Figure 6A and B, *P <* 0.001) or the serum starvation condition (Figure 6C and D, *P <* 0.001). These results combined with observations stated above suggested that miR-645, up-regulated in human AGEJ tissues, inhibited cell apoptosis and promotes tumorigenicity via suppressing caspase-3/7 activity by targeting IFIT2.

## Discussion and conclusions

Although accumulating evidence have shown that miRNAs deregulation is involved with tumor carcinogenesis, progression, migration and invasion [41], metastasis [42, 43] and multidrug resistance [44–46]. Little is known about the roles of miRNAs in the development of adencarcinoma of gastric esophageal junction (AGEJ). Here, we showed that that miR-645 expression was significantly increased in AGEJ clinical specimens compared with paired non-cancerous tissues and was significantly up-regulated in two gastric cancer (GC) cell lines, SGC7901 and BGC-823, which were used alternative cell models because no available AGEJ cell lines were established to date. Inhibition of miR-645 in SGC7901 and BGC-823 cells significantly induced apoptosis of SGC7901 and BGC-823 cells in the condition of serum starvation or chemotherapeutic drug by up-regulating *IFIT2,* a mediator of apoptosis, with a potential binding site for miR-645 in its mRNA’s 3′UTR. The expression pattern of miR-645 and IFIT2 in SGC7901 and BGC-823 cells and clinical samples were negatively correlated, further suggesting that *IFIT2* is a target gene of miR-645. Moreover, inhibition of miR-645 results in increased caspase-3/7 activity, which is activated by IFIT2. All these findings suggest a fundamental role of miR-645 in carcinogenesis, especially in the development of AGEJ.

Too little apoptosis is one crucial cause of carcinogenesis because malignant cells death are reduced remarkably [47, 48], resulting in malignant transformation of the affected cells, tumour metastasis and multidrug resistance of cancer cells. Hence, apoptosis is of great importance in the treatment of cancer and is a popular target of many treatment strategies. In this study, we showed that miR-645 impaired cancer cells to serum deprivation–induced apoptosis, whereas the depletion of miR-645 antagonized this effect of miR-645, suggesting that miR-645 may play a crucial role in the adaptation of cancer cells to low nutrition. Increasing numbers of miRNAs have been implicated in the cancer cell apoptosis. On the one hand, microRNAs might function as tumor suppressor via inducing apoptosis, i.e. miR-421, which induces cell proliferation and apoptosis resistance in human nasopharyngeal carcinoma via down-regulation of FOXO4 [49]; miR-149, which induces apoptosis by inhibiting Akt1 and E2F1 in human cancer cells [50] and miRNA-31, which induces apoptosis in human neuroblastoma cells [51]. On the other hand, microRNAs might function as oncogenes by suppressing apoptosis, i.e. miR-24, which inhibits apoptosis and represses Bim in mouse cardiomyocytes [52]; miR-886-5p, which inhibits apoptosis by down-regulating Bax expression in human cervical carcinoma cells [53], and miR-183, which inhibits TGF-β1-induced apoptosis by downregulation of PDCD4 expression in human hepatocellular carcinoma cells [54].

ISGs, IFN stimulated genes, refer to genes that are tanscribed by IFNs induction. Among them, 4 can play important roles that affect both the inhibition of viral replication and the inhibition of cellular proliferation [55, 56]. These genes can inhibit viral replication by sacrificing the cell through promoting apoptosis and suppress the cancer progression via inhibiting the malignantly transformed cell survival for the benefit of the host [57]. The ISG54 gene codes for a protein of 54 kDa (472 aa) with tetratricopeptide repeats (TPR) and has also been designated IFN-induced protein with tetratricopeptide repeats 2(IFIT2) [58–60]. It is one of four related human ISGs with characteristic TPR motifs. ISG54 (IFIT2) functions as a mediator of apoptosis [60]. In our study, we observed a significant down-regulation of IFIT2 in AGEJ tissues compared with paired non-cancerous tissues, moreover, bioinformatics analysis and luciferase reporter assay indicated that IFIT2 is one target of miR-645. Hence, we assume that over-expression of miR-645 might lead to down-regulation of IFIT2 and in turn the resistance of cells to apoptosis, resulting in AGEJ progression.

Reports have shown that the activation of caspase-3, a key mediator of the execution phase of apoptosis, was clearly apparent in cells expressing ISG54. Pathways leading to caspase activation and apoptosis are often designated as either extrinsic or intrinsic [61]. The extrinsic pathway initiates outside the cell by transmembrane death receptors and the subsequent activation of caspases [61]. The intrinsic pathway, also called the mitochondrial pathway, is dependent on pro-apoptotic proteins such as Bax or Bak that induce mitochondrial outer membrane permeability, release of apoptotic molecules, and activation of caspases [62]. In the present study, we examined the capase-3/7 activity following miR-645 depletion and IFIT2 expression treatment to find that miR-645 expression down-regulation led to up-regulation of IFIT2 and increased capase-3/7 activity, suggesting the role of miR-645 promoting cancer progression via suppressing transformed cell apoptosis through inhibiting IFIT2 expression and capase-3/7 activity.

In summary, our data indicate that miR-645 may function as an oncogene in tumorigenicity of adencarcinoma of gastric esophageal junction and has an important role in inhibiting IFIT2, hence, the up-regulation of miR-645 inhibits the AGEJ cells apoptosis. Moreover, our results showed that IFIT2 may act as a tumor suppressor in the development of AGEJ. However, owing to the fact that each miRNA may regulate many target genes which can affect carcinogenesis in different ways, more studies are needed to investigate other miR-645 targets which may have critical roles in AGEJ tumorigenesis. The present study also provides novel insights into the role of miR-645 in human AGEJ and indicates that miR-645 may serve as a therapeutic target of AGEJ.

## Electronic supplementary material

Additional file 1: Table S1: Antibodies used in western blotting assay (DOCX 15 KB)

Additional file 2: Figure S1: miR-645 expression of SGC7901 and BGC-823 was significantly up-regulated compared with immortalized GC cell line, GES-1. miR-645 expression level in SGC7901 and BGC-823 were 6.9 and 4.4 - fold higher than in GES-1 (One-way ANOVA analysis, F = 129.393, ***P < 0.001). (TIFF 641 KB)

Additional file 3: Figure S2: Wild-type and mutant *IFIT2*-3′UTR containing the putative binding site of miR-645 were cloned into psiCHECK-2 vector. A. *IFIT2*-3′UTR was amplified from genomic DNA of SGC7901. B. Lane 1 & 3: Recombinant plasmids of *IFIT2-1, IFIT2-2* respectively; lane 2 & 4: Results of enzyme digestion of recombinant plasmids of *IFIT2-1*and *IFIT2-2* respectively. Results showed that *IFIT2*-1/2 have been successfully inserted into the vectors (M1: DL2000 DNA Marker; M2: DL1 kb DNA Marker; ZTBT2-1/2 bands: 1902 bp; Vectors bands: 6.1 Kb). C. M1: 1 kb DNA Ladder Marker. Lane 1: amplification of mut*IFIT2* PCR1. One band of mut*IFIT2* (8.1 Kb) demonstrated the successful PCR of mutant amplification. D. Sequencing data of WT-*IFIT2* and MT –*IFIT2*. (TIFF 3 MB)

## Abbreviations

miRNA: microRNA
AGEJ: adencarcinoma of gastric esophageal junction
GC: gastric cancer.
