# Supplementary material for: MicroRNA-645, up-regulated in human adencarcinoma of gastric esophageal junction, inhibits apoptosis by targeting tumor suppressor IFIT2
Source: BMC Cancer. 2014 Aug 29;14:633. doi: 10.1186/1471-2407-14-633 (PMC4161885; doi:10.1186/1471-2407-14-633)
Supplement: Supplementary file 1 — Additional file 1: Table S1: Antibodies used in western blotting assay (DOCX 15 KB) [file 12885_2013_4823_MOESM1_ESM.docx]

| Antibodies used in western blotting assay | | | | |
| --- | --- | --- | --- | --- |
| Antibody name | **Producer/product code** | **Molecular weight** | **Origin** | **dilution** |
| IFIT2 | Abcam/ab55837 | 54 kDa | Rabbit | 1:1000 |
| Tublin | Abcam/ab6046 | 50 kDa | Rabbit | 1:500 |
