# Supplementary figures and images for: MicroRNA-645, up-regulated in human adencarcinoma of gastric esophageal junction, inhibits apoptosis by targeting tumor suppressor IFIT2
Source: BMC Cancer. 2014 Aug 29;14:633. doi: 10.1186/1471-2407-14-633 (PMC4161885; doi:10.1186/1471-2407-14-633)

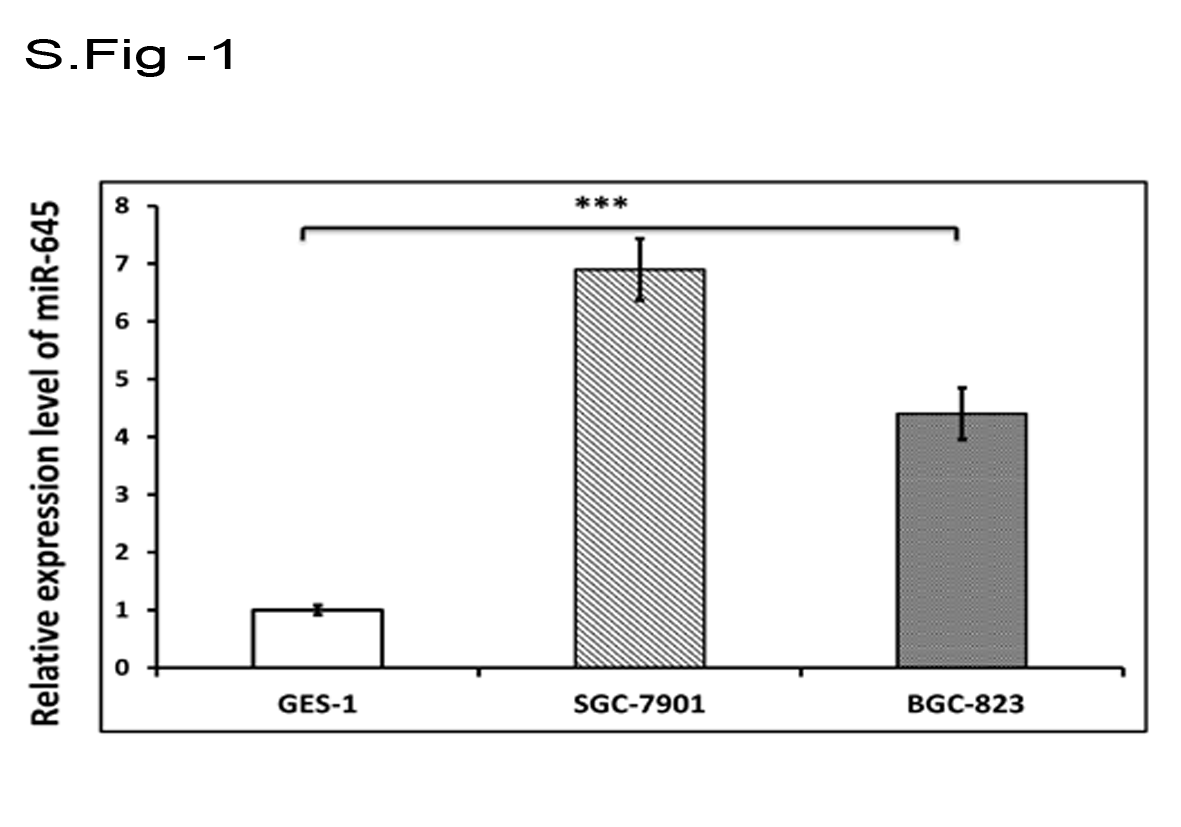

Supplement: Supplementary file 2 — Additional file 2: Figure S1: miR-645 expression of SGC7901 and BGC-823 was significantly up-regulated compared with immortalized GC cell line, GES-1. miR-645 expression level in SGC7901 and BGC-823 were 6.9 and 4.4 - fold higher than in GES-1 (One-way ANOVA analysis, F = 129.393, ***P < 0.001). (TIFF 641 KB) [file 12885_2013_4823_MOESM2_ESM.tiff]

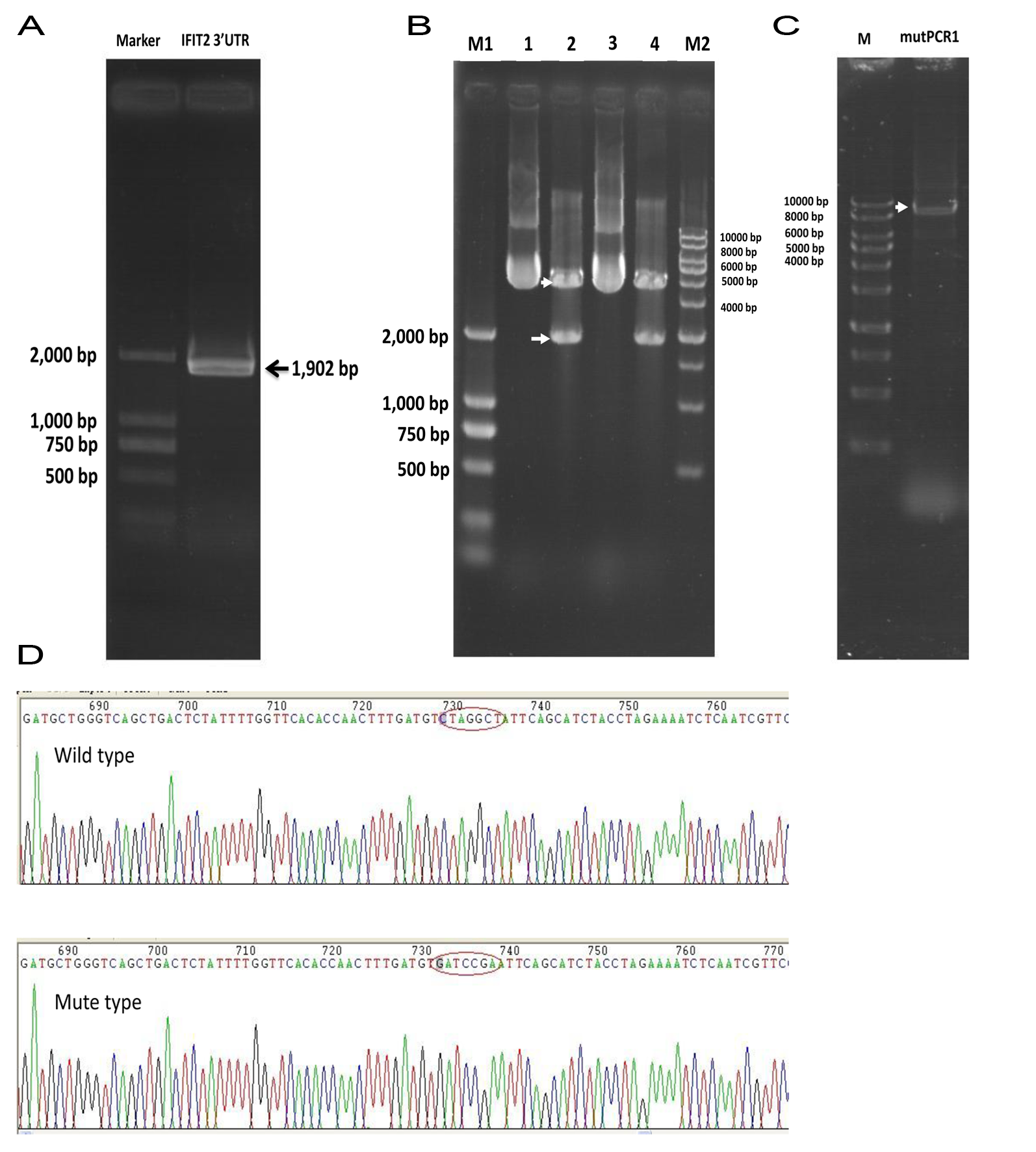

Supplement: Supplementary file 3 — Additional file 3: Figure S2: Wild-type and mutant IFIT2-3′UTR containing the putative binding site of miR-645 were cloned into psiCHECK-2 vector. A. IFIT2-3′UTR was amplified from genomic DNA of SGC7901. B. Lane 1 & 3: Recombinant plasmids of IFIT2-1, IFIT2-2 respectively; lane 2 & 4: Results of enzyme digestion of recombinant plasmids of IFIT2-1and IFIT2-2 respectively. Results showed that IFIT2-1/2 have been successfully inserted into the vectors (M1: DL2000 DNA Marker; M2: DL1 kb DNA Marker; ZTBT2-1/2 bands: 1902 bp; Vectors bands: 6.1 Kb). C. M1: 1 kb DNA Ladder Marker. Lane 1: amplification of mutIFIT2 PCR1. One band of mutIFIT2 (8.1 Kb) demonstrated the successful PCR of mutant amplification. D. Sequencing data of WT-IFIT2 and MT –IFIT2. (TIFF 3 MB) [file 12885_2013_4823_MOESM3_ESM.tiff]
